# Inhibition of adipose triglyceride lipase (ATGL) by the putative tumor suppressor G0S2 or a small molecule inhibitor attenuates the growth of cancer cells

**DOI:** 10.18632/oncotarget.5061

**Published:** 2015-07-31

**Authors:** Rachid Zagani, Wissal El-Assaad, Isabelle Gamache, Jose G. Teodoro

**Affiliations:** ^1^ Goodman Cancer Research Centre, McGill University, Montréal, QC, Canada; ^2^ Department of Biochemistry, McGill University, Montréal, QC, Canada

**Keywords:** lipase, ATGL, G0S2, metabolism, lipids, triglycerides, tumor suppressor

## Abstract

The G0/G1 switch gene 2 (*G0S2*) is methylated and silenced in a wide range of human cancers. The protein encoded by *G0S2* is an endogenous inhibitor of lipid catabolism that directly binds adipose triglyceride lipase (ATGL). ATGL is the rate-limiting step in triglyceride metabolism. Although the *G0S2* gene is silenced in cancer, the impact of ATGL in the growth and survival of cancer cells has never been addressed. Here we show that ectopic expression of G0S2 in non-small cell lung carcinomas (NSCL) inhibits triglyceride catabolism and results in lower cell growth. Similarly, knockdown of ATGL increased triglyceride levels, attenuated cell growth and promoted apoptosis. Conversely, knockdown of endogenous *G0S2* enhanced the growth and invasiveness of cancer cells. G0S2 is strongly induced in acute promyelocytic leukemia (APL) cells in response to all trans retinoic acid (ATRA) and we show that inhibition of ATGL in these cells by G0S2 is required for efficacy of ATRA treatment. Our data uncover a novel tumor suppressor mechanism by which G0S2 directly inhibits activity of a key intracellular lipase. Our results suggest that elevated ATGL activity may be a general property of many cancer types and potentially represents a novel target for chemotherapy.

## INTRODUCTION

Reprogramming of metabolic networks is an emerging hallmark of cancer cells [1]. For example, enhanced glycolysis (Warburg effect), is a very general property of tumor cells [2]. Reprogramming of lipid metabolism is not as well understood although several studies suggest that these pathways are also extensively altered in cancer cells. Lipogenesis is markedly increased in cancer cells [3-9]. Enzymes of lipid biosynthesis, including ATP-citrate lyase, fatty acid synthase, acetyl-CoA carboxylase-1, dicarboxylate transporter and malic enzyme are upregulated in many cancer types and abrogation of their activity often leads to apoptosis and/or growth arrest [3-9].

Fatty acids (FA) in most tissues are first converted to triglycerides (TGs) and stored in specialized organelles called lipid droplets. TGs are synthesized by the condensation of three fatty-acyl moieties to glycerol. The hydrolysis and mobilization of FA from TGs, is controlled by specific lipases in the triglyceride/free fatty acid (TG/FFA) cycle. The TG/FFA cycle is a coordinated cycle of lipid catabolism breaking down TGs stepwise into FFAs and glycerol. The intracellular lipolytic pathway is regulated by a series of enzymes each responsible of hydrolyzing a specific lipid molecule. Adipose triglyceride lipase (ATGL), initiates the process of TG metabolism by hydrolyzing TGs into diacylglycerol (DAG) and FA [10-12]. The subsequent step requires hormone sensitive lipase (HSL) which breaks down DAG into monoacylglycerol (MAG) and FA [13]. Finally, MAG is further broken down into FA and glycerol by monoacylglycerol lipase (MAGL) [14].

The TG/FFA cycle appears to be accelerated in cancer with activity of both the synthetic and catabolic arms becoming elevated. MAGL activity was recently shown to be dramatically upregulated in human cancer which rendered tumor cells highly aggressive [15]. MAGL expression is elevated several fold in ductal breast cancer cells and was shown to play a role in the epithelial-to-mesenchymal transition of prostate cancer cells [16]. MAGL regulates a network of oncogenic signaling lipids, which likely promote migration, invasion, and tumour growth [15, 17]. MAGL was also shown to be essential for the proliferation and tumorigenicity of colorectal cancer cells as either its inhibition or deletion led to attenuated tumour growth and cancer cell proliferation [18]. Although the importance of elevated MAGL activity in cancer has been demonstrated, the rate-limiting enzyme of TG catabolism is ATGL and thus far no mechanism has been proposed suggesting how this initiating enzyme in TG lipolysis becomes elevated in cancer.

The G_0_/G1 switch gene 2 (*G0S2*) encodes an endogenous inhibitor of ATGL [19]. The name *G0S2* derives from the fact that it was initially identified in monocytes as a gene upregulated during transition from G_0_ to G1 phases of the cell cycle [20, 21]. The *G0S2* gene encodes a small 12kDa protein that localizes to the mitochondria and endoplasmic reticulum [22, 23] and is expressed in most tissues, with the highest levels in adipose tissues and liver [19]. G0S2 directly inhibits lipase activity by interacting with the N-terminal patatin domain of ATGL [24]. Three properties of G0S2 suggest that the protein functions as a tumor suppressor. First, the *G0S2* gene has a potent CpG island in the promoter region [20] and work from several groups have demonstrated that the gene is silenced in many types of human cancer including head and neck cancer [25], glioma [26] lung [27, 28] and breast cancer [22]. Second, ectopic expression of *G0S2* in a variety of human tumor cells promotes cell death [22] and can also inhibit proliferation of hematopoietic stem cells and CML [29, 30]. Lastly, knockdown of *G0S2* expression in primary mouse embryo fibroblasts was shown to enhance oncogene-induced cell transformation [22]. Although G0S2 has the properties of a tumor suppressor, it has never been determined if ATGL inhibition is required for G0S2 mediated suppression of cell growth.

In the current study we show that the tumor suppressor properties of G0S2 are derived at least in part from its capacity to inhibit ATGL. Inhibition of ATGL by G0S2, RNAi, or a small molecule inhibitor was able to attenuate the growth and motility of tumor cells. These data show that *G0S2* encodes a tumour suppressor protein that links regulation of lipid catabolism to cell transformation and suggests that ATGL may be a novel target to limit growth of tumour cells.

## RESULTS

### Ectopic expression of *G0S2* results in elevated cellular TG levels and inhibits the growth, survival and motility of cancer cells

**Figure 1:**
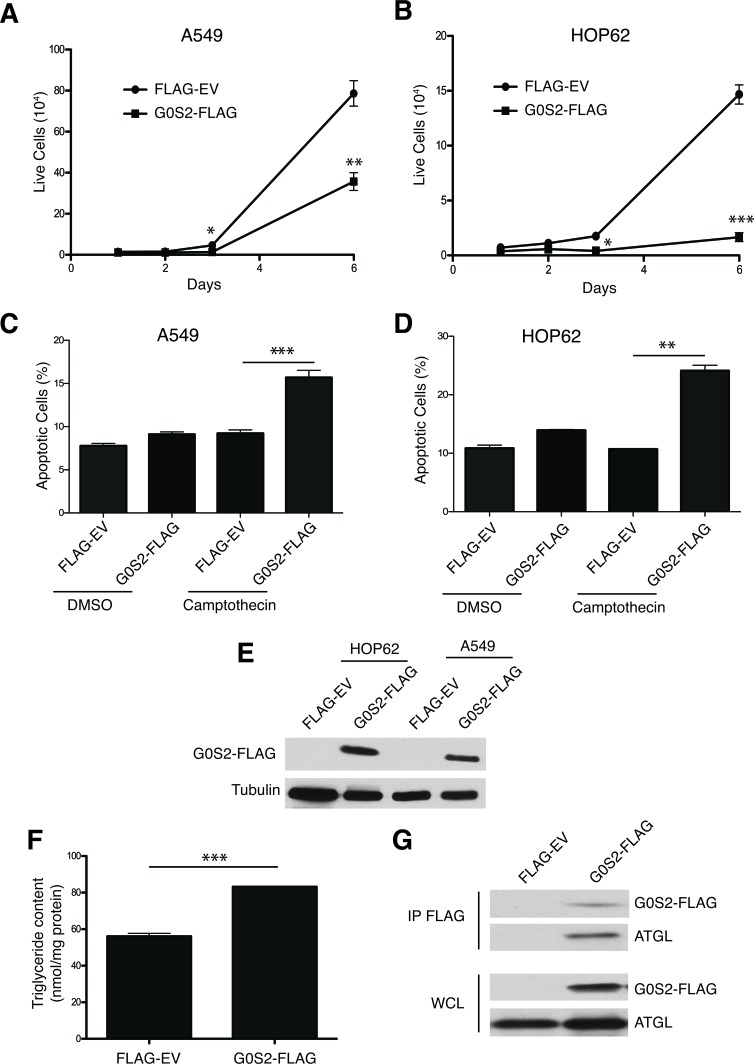
Ectopic expression of G0S2 results in elevated cellular TG levels and inhibits the growth, survival and motility of cancer cells **A.**-**B.** Growth curves of polyclonal populations of A549 (panel A) and HOP62 (panel B) cells stably transduced with either pBABE Flag-tagged G0S2 (G0S2-FLAG) or empty vector (Flag-EV) retrovirus. Equal number of cells stably expressing G0S2-Flag or Flag-EV were seeded in a 12 well plate and counted on the indicated days. **C.**-**D.** A549 (panel C) or HOP62 (panel D) cells stably expressing G0S2 or empty pBABE were treated for 24h with camptothecin (10 um) or vehicle control. Cell death was monitored by flow cytometry using Annexin V and 7AAD staining. The percentage of apoptotic cells (Annexin-positive) is indicated. **E.** Whole cell lysates from HOP62 and A549 stable cell populations were separated by SDS-PAGE and levels of G0S2 were determined by immunoblotting with anti-FLAG antibody. **F.** A549 cells stably expressing G0S2-FLAG or empty vector were incubated in 300 μM oleic acid for 5 hr and the intracellular triglyceride content was determined 4 hours later. The data were normalized to protein concentration in the cell extracts. **G.** A549 cells stably expressing G0S2-FLAG or empty vector were lysed and cell extracts were immunoprecipitated using anti-FLAG antibody. IPs and whole cell lysates (WCL) were analyzed by immunoblotting using antibodies to detect G0S2 (FLAG) and endogenous ATGL.

G0S2 has the general properties of a tumor suppressor protein and appears to play a major role in lipid metabolism by binding ATGL and suppressing lipase activity [31-33]. It is not known if the growth inhibitory properties of G0S2 stem from its ability to inhibit ATGL or other functions. To further study the tumor suppressor activity of G0S2, non-small cell lung carcinoma (NSCL) cell lines that stably express G0S2 were generated. NSCL cells were selected as a model since the *G0S2* gene was shown to be methylated and silenced in this cancer type and re-expression of the gene was shown to induce death [22, 27, 28]. A549 and HOP62 cells were transduced with retrovirus expressing either FLAG-tagged G0S2 or empty vector controls (EV). Figure 1A and 1B show that G0S2 expression resulted in slower growth in both A549 and HOP62 lines. In addition to slow growth, G0S2 expressing cell lines also displayed greater sensitivity to the chemotherapy agent Camptothecin (Figure 1C and 1D). Expression of FLAG-G0S2 in the cell lines was confirmed by western blot analysis using anti-FLAG antibody (Figure 1E).

In order to determine if expression of G0S2 was able to inhibit the lipase activity of ATGL, A549 cells were loaded with oleic acid for 5 hours and total cellular triglyceride (TG) levels were measured and normalized to total protein. Figure 1F shows that A549 cells expressing G0S2 maintained significantly more TG relative to EV control. G0S2 was previously shown to directly interact with ATGL in metabolic tissues such as adipose tissue and liver [19]. In Figure 1G we show using co-immunoprecipitation that FLAG-G0S2 also forms a complex with endogenous ATGL in A549 cells. These data show that re-expression of G0S2 in NSCL cell lines, which is commonly methylated and silenced in these cells, results in slow growth, increased susceptibility to apoptosis and accumulation of TG through ATGL inhibition.

### Knockdown of *G0S2* enhances cell growth and motility in colorectal cancer cells

**Figure 2:**
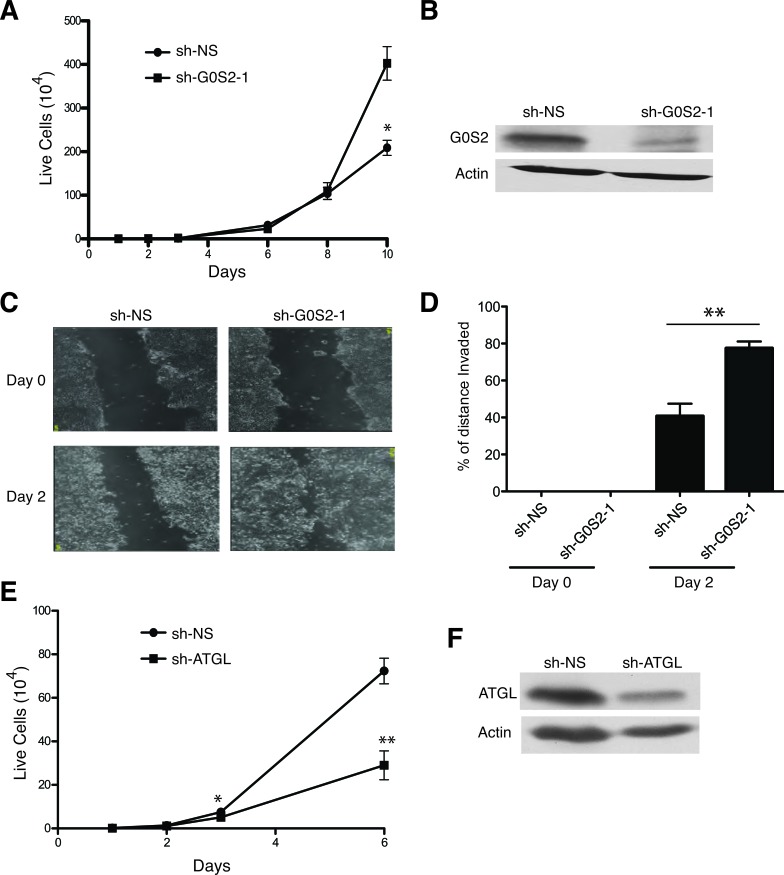
Knockdown of G0S2 enhances cell growth and motility in colorectal cancer cells **A.** Growth curves of HT29 transduced with lentiviral vectors encoding an shRNA targeting G0S2 (sh-G0S2) or a non-silencing control (sh-NS). Cells were seeded in a 12 well plate and counted on the indicated days. **B.** shG0S2 and sh-NS cells were analyzed by immunoblotting to detect the expression level of endogenous G0S2 protein in whole cell lysates. β-actin was used as a loading control. **C.** *In vitro* scratch assay using sh-G0S2 and sh-NS cells showing images captured at 0 and 2 days after scratching. **D.** Quantitation of motility from the scratch assays shown in panel C. Motility was calculated as described in the materials and methods. **E.** Growth curve of HT29 cells stably transduced with an shRNA targeting ATGL or sh-NS. **F.** ATGL protein expression was analyzed in whole cell lysates by immunoblotting with anti-ATGL antibody. β-actin was used as a loading control.

The study of endogenous G0S2 in cancer cells is complicated by the fact that the gene is highly methylated and silenced in most tumor-derived cell lines. Although we were unable to detect G0S2 protein in NSCL cell lines, the colorectal cell line HT29 did show an appreciable immunoblot signal for G0S2. Figure 2A shows that when expression of G0S2 is silenced in HT29 cells using a stable shRNA, cell growth is significantly increased. Expression and knockdown of G0S2 was confirmed by immunoblot against endogenous G0S2 (Figure 2B). In addition to cell growth, the motility of HT29 G0S2 knockdown cells was also enhanced as measured using scratch assays (Figure 2C and 2D).

Since G0S2 is thought to exert effects on lipid metabolism by binding and inhibiting ATGL, we hypothesized that knockdown of ATGL would have similar biological effects as ectopic expression of G0S2. Figure 2E shows that knockdown of ATGL using a stable shRNA hairpin significantly inhibited the growth of HT29 cells. Knockdown of ATGL was confirmed by western blot analysis shown in Figure 2F. These data indicate that elevated ATGL activity in tumor cells enhances the growth properties of the cells and that inhibition of ATGL by either expression of G0S2 or knockdown of ATGL results in slower cell growth.

### ATGL knockdown inhibits cell growth, motility and promotes apoptosis

**Figure 3:**
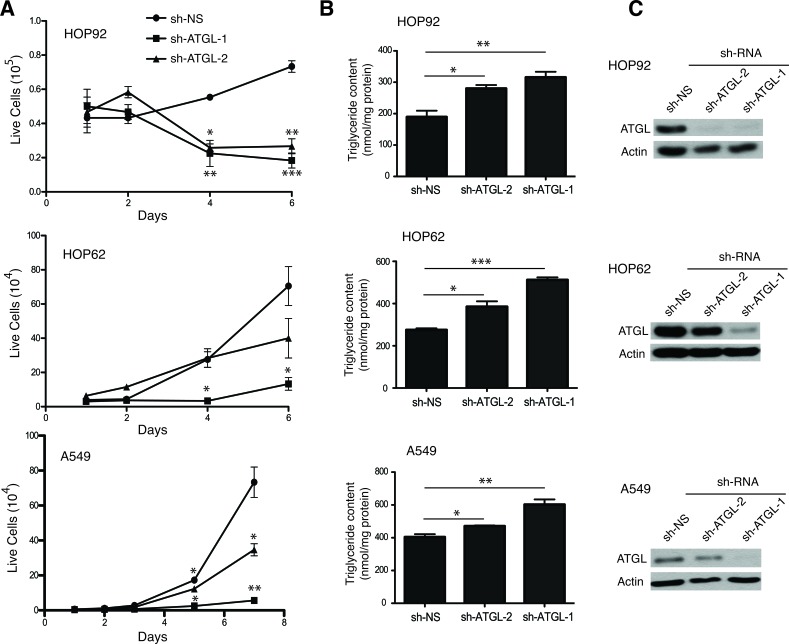
ATGL knockdown inhibits cell growth of NSCL cells **A.** Growth curves of HOP-92, HOP-62 and A549 cells transduced with lentivirus expressing shRNAs targeting ATGL or non-silencing control (shRNA-NS). **B.** Polyclonal populations of ATGL knockdown or control cells were incubated with 400 μM of oleic acid for 6h and the triglyceride level measured 4 hours later as described in the materials and methods. **C.** ATGL protein expression was analyzed in whole cell lysates by immunoblotting with anti-ATGL antibody. Tubulin was used as a loading control.

**Figure 4:**
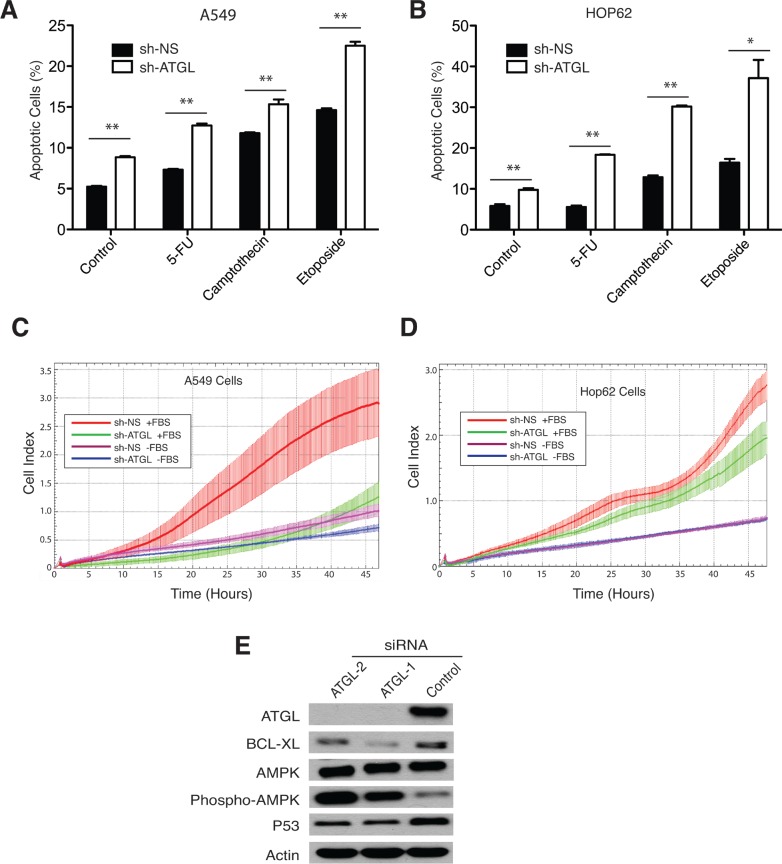
ATGL knockdown promotes apoptosis and inhibits migration of NSCL cancer cells **A.**-**B.** The NSCL cancer cell lines A549 (panel A) and HOP62 (panel B) stably expressing an shRNA hairpin targeting ATGL (sh-ATGL) or non-silencing control (shRNA-NS) were seeded in 6-well plates and incubated for 24h with the DNA damaging-agents, 5-FU (100 μM), Etoposide (25 μM), Camptothecin (10 μM) or vehicle control. Apoptosis was measured by flow cytometry using Annexin V/7AAD staining. The percentage of Annexin V positive cells is indicated for each treatment. **C.**-**D.** A modified Boyden chamber assay monitored by xCELLigence real-time cell analysis was used to measure serum-mediated migration in ATGL knockdown and control cells. Experiments were performed in the presence or absence of fetal bovine serum (FBS) to confirm the dependence of motility on serum. **E.** Knockdown of ATGL was performed using 2 different siRNA duplexes or a non-silencing control. Immunoblotting was used to confirm ATGL knockdown and to detect variations in BCL-XL, AMPK, phospho-AMPK and p53. Actin was used as a loading control.

Since G0S2 overexpression resulted in elevated TG levels in A549 and HOP62 cells, we hypothesized that ATGL inhibition may explain some of the tumor suppressor properties displayed by G0S2 in NSCL. In order to test this hypothesis we determined if knockdown of ATGL expression using RNAi affected the growth properties of NSCL carcinoma cell lines. Figure 3A shows that stable expression of short hairpin RNAs (shRNA) inhibiting ATGL expression significantly inhibited the growth of three different NSCL lines (HOP92, HOP62 and A549) relative to non-silencing (NS) control. In order to confirm that ATGL enzymatic activity was being affected in the knockdown cells, total TG levels were measured and normalized to total protein. Figure 3B shows that TG levels were significantly elevated in ATGL knockdown cells indicating that the capacity of ATGL to hydrolyze TG in these cells was compromised. Knockdown of ATGL in these cells was confirmed by western blot (Figure 3C). Since we previously observed that expression of G0S2 could induce cell death or sensitize cells to killing by chemotherapeutic agents [22], we tested if ATGL knockdown cells were similarly affected. Figure 4A and 4B shows that knockdown of ATGL in NSCL cell lines sensitized them to cell death induced by 5-fluorouracil, camptothecin or etoposide. We then determined if knockdown of ATGL could also affect the motility of NSCL cells. A modified Boyden chamber assay was used in which the xCELLigence system monitored serum-mediated motility in real-time. Figure 4C and 4D showed that knockdown of ATGL in HOP62 and A549 cells resulted in reduced motility. A recent study suggested that knockdown of ATGL in colorectal cancer cells resulted in elevated phospho-AMPK and p53 [34]. Figure 4E shows that knockdown of ATGL in A549 cells also resulted in elevated phospho-AMPK suggesting that the mechanism of slow growth may be due to activation of the p53 pathway through AMPK. Taken together, these data show that inhibition of activity of the intracellular lipase ATGL can have a significant impact on the growth, motility and sensitivity to cell death of NSCL cells.

### A chemical inhibitor of ATGL suppresses the growth of tumor cells

**Figure 5:**
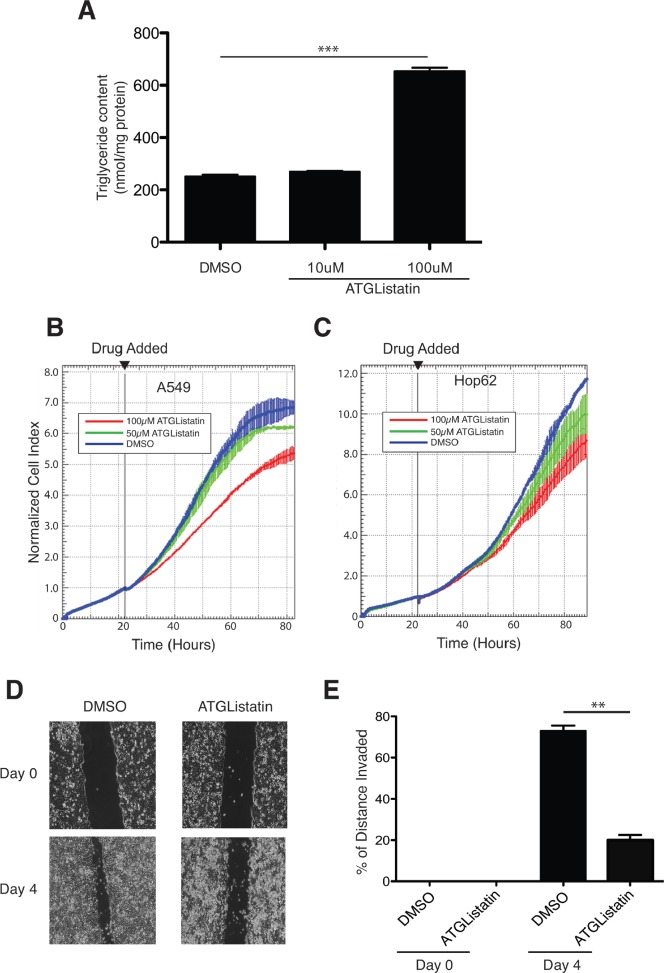
A chemical inhibitor of ATGL, Atglistatin, suppresses the growth of cancer cells **A.** A549 cells were plated in 10 cm plates and cultured under standard conditions for 24 hours. The next day, cells were treated with the indicated concentrations of Atglistatin or vehicle control (DMSO) for another 24 hours. Media were replaced with fresh media containing 400 μM of oleic acid and TG levels were measured after 4 hours as described in the materials and methods and. TG levels were normalized to the total protein in the cell extracts. **B.**-**C.** xCELLigence real-time cell analysis of A549 (panel A) and HOP62 (panel B) cells measured in the presence of the indicated concentrations of Atglistatin or vehicle control (DMSO). Time at which drug was added is indicated (22 hours after plating). **D.** *In vitro* scratch assay using Atglistatin or DMSO (vehicle) treated HT29 cells showing images captured at 0 and 4 days after scratching. **E.** Quantitation of motility from the scratch assays shown in panel D. Motility was calculated as described in the materials and methods.

Findings from several groups demonstrated that expression of G0S2 is silenced across a wide-range of tumor types. Since G0S2 acts as an endogenous inhibitor of ATGL activity, it is possible that elevated ATGL activity is a general property of cancer cells and thus represents an attractive target for therapeutic intervention. Recently, a highly specific inhibitor of ATGL activity was developed termed Atglistatin that was shown to be effective both *in vivo* and *in vitro* [35]. Atglistatin was shown to be a highly selective inhibitor for ATGL without affecting other intracellular lipases such as MAGL or HSL. As expected, treatment with Atglistatin also resulted in the accumulation of TG in cells confirming that enzymatic activity of ATGL was being effectively inhibited (Figure 5A). We therefore determined if Atglistatin was also able to inhibit the growth of NSCL lines. Figure 5B and 5C show that Atglistatin was able to inhibit the growth of A549 and HOP62 cells in a concentration dependent manner. Since we showed in Figure 2 that G0S2 could inhibit the motility of HT29 cells, we performed the same assay using Atglistatin. Figure 5D and 5E show that Atglistatin was able to inhibit the motility of HT29 cells using a scratch assay. These results show that a specific small molecule inhibitor of ATGL can slow the growth of cancer cells and that ATGL is a potential target for chemotherapy.

### ATGL inhibition by G0S2 partially mediates the therapeutic effects of all-trans retinoic acid in acute promelocytic leukemia

**Figure 6:**
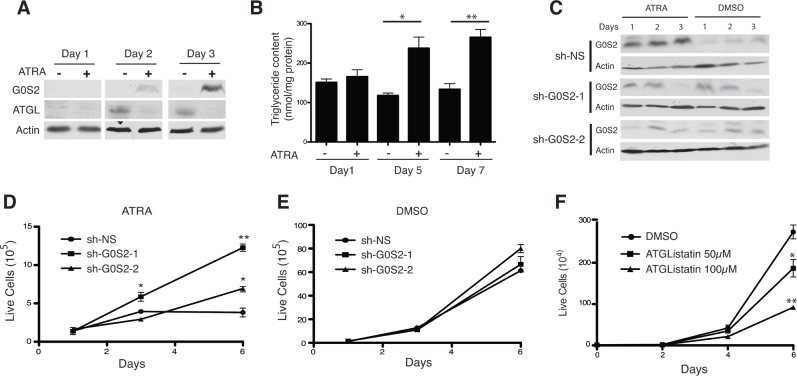
ATRA-mediated growth arrest depends on upregulating G0S2 in NB4 cells **A.** NB4 cells were treated with 1 μM ATRA and at the indicated time points equal amounts of total cell lysates were immunoblotted with anti-G0S2 and anti-ATGL. β-actin was used as a loading control. **B.** NB4 cells were incubated in 1 μM ATRA for the indicated times and the intracellular triglyceride content was determined as described in the materials and methods and. The data were normalized with total protein amounts in the cell extracts. **C.** NB4 cells stably expressing shRNAs targeting G0S2 (sh-G0S2) or non-silencing (sh-NS) control were cultured in 1μM ATRA or vehicle control (DMSO). Knockdown efficiency was examined in whole-cell lysates by immunoblotting with anti-G0S2. β-actin was used as a loading control. **D.**-**E.** Growth curves of sh-G0S2 and sh-NS NB4 cells in the presence of 1μM ATRA (panel D) or vehicle control (panel E). **F.** Growth curves of NB4 cells treated with the indicated concentrations of Atglistatin or vehicle control (DMSO).

A previous study has shown that *G0S2* mRNA and protein is strongly induced in response to treatment with all-*trans* retinoic acid (ATRA) in Acute Promyelocytic Leukemia (APL) cells [36]. ATRA is the frontline therapy for APL and the induction of *G0S2* expression in this system suggests that the tumor suppressor properties of the protein may play a role in the response to the drug. We confirmed previous observations showing that treatment of the APL cell line NB4 with ATRA results in the potent induction of G0S2 protein over three days (Figure 6A). Intriguingly, whereas levels of G0S2 are elevated with ATRA treatment, we observed ATGL levels are decreased. This suggests that suppression of TG metabolism is an important process in the differentiation program induced by ATRA. Endogenous TG levels in NB4 cells treated with ATRA were significantly elevated after 5 and 7 days of ATRA treatment relative to DMSO treated-controls (Figure 6B). This result indicates that G0S2 induction in NB4 cells resulted in inhibition of ATGL activity as shown by increased TG levels.

In order to determine if G0S2 expression plays a role in the response of NB4 cells to ATRA, we transduced these cells with two different lentivirus vectors encoding shRNAs targeting G0S2. Figure 6C shows that both shRNA constructs were able to attenuate the induction of G0S2 in response to ATRA. Interestingly, NB4 cells in which *G0S2* is silenced were significantly more resistant to ATRA-induced growth arrest than control cells expressing a non-silencing control (Figure 6D and 6E) suggesting that G0S2 is required at least in part for the inhibition of cell growth in response to ATRA. Furthermore, treatment of NB4 cells with Atglistatin in the absence of ATRA was also able to inhibit NB4 cell growth (Figure 6F). Taken together these data show that the induction of G0S2 expression during ATRA differentiation therapy of PML inhibits TG catabolism and cell growth.

## DISCUSSION

The *G0S2* gene has the general properties of a tumour suppressor and has been shown to be a potent negative regulator of triglyceride catabolism. In the current study we show for the first time that the inhibition of ATGL by G0S2 can significantly reduce the growth and motility of a variety of tumour cell lines. Furthermore, inhibition of ATGL using either RNAi or a chemical inhibitor can similarly slow the growth of tumor cells. Since G0S2 expression is reduced in such a wide range of cancer cell types, this implies that elevated ATGL activity may be a common property of cancer cells and may represent a novel target for cancer therapy. These observations also highlight the growing importance of altered lipid metabolism in the process of cell transformation and cancer.

The reason(s) why inhibition of intracellular lipase activity may interfere with cell division have yet to be fully explained. A recent paper demonstrated that Monoacylgycerol lipase (MAGL) activity is upregulated in some cancer cells thus rendering tumors derived from these cells more aggressive [15]. In the same study, elevated MAGL activity was shown to increase production of fatty acid derived signaling molecules, which in turn enhances cell growth and motility. MAGL is the last step in TG catabolism resulting in glycerol and non-esterified fatty acids as final products. Since ATGL is the first step in TG catabolism, it is tempting to speculate that observed increases in MAGL activity may be due to loss of G0S2 in cancer cells, leading to enhanced ATGL activity by generating substrates for MAGL. An obvious explanation for reduced cell growth caused by ATGL inhibition would simply be the loss of a potent energy source. The intense energy demands of rapidly dividing cells may necessitate tapping the dense energy pools stored in triglycerides and G0S2 may function as a checkpoint preventing access to energy supplied by oxidation of fatty acids.

Study of the yeast ortholog of ATGL (Tgl4) has shown that lipase activity of Tgl4 is required for proper cell cycle progression. Tgl4 activity was shown to be activated through phosphorylation by the cyclin-dependent kinase Cdk1/Cdc28 (ortholog of mammalian Cdc2). Interestingly, mutation of the Cdk1 phosphorylation sites on Tgl4 resulted in delayed cell cycle entry [37]. It remains to be determined if lipolysis in mammals is under similar cell cycle control, however, examination of the primary amino acid sequence of mammalian ATGL reveals that cdk1 consensus sites exist at serine 185 and threonine 489. In the case of yeast, activation of lipase activity occurs at the G1/S transition of the cell cycle, which coincides with bud emergence, which requires increased amounts of fatty acids. Yeast does not appear to have a homolog of G0S2 but the requirement for precise regulation of ATGL-like activity during the cell cycle establishes an intriguing precedent and it will be interesting to determine if similar regulation occurs in mammals.

Our results showing that ATGL knockdown can inhibit cell growth is in accordance with a recent study by Ou et al. who showed that knockdown of ATGL in HCT116 colorectal cancer cells resulted in a slow growth phenotype and elevated phospho-AMPK and p53 [34]. Intriguingly, in the same study the authors observed that knockdown of the ATGL co-activator, cgi-58 (also known as Abhd5) resulted in cellular transformation and enhanced growth and cell motility. The opposite phenotype of cgi-58 knockdown suggests that this protein has tumour suppressor effects that are independent of ATGL.

A recent study demonstrated that G0S2 is able to suppress the growth of the chronic myeloid leukemia (CML) cell line, K562 both *in vitro* and in mouse xenografts [30]. Like in many other tumor cells, the promoter of G0S2 was shown to be methylated in K562 cells is reversed using the methylation inhibitor 5-Azacytadine (5-Aza). In this study the authors propose that the mechanism of action of G0S2 was through interaction with nucleolin, which caused the retention of nucleolin in the cytosol and inhibits its growth promoting functions. The same group also demonstrated that G0S2 was able to maintain quiescence of hematopoetic stem cells also through interaction with nucleolin [29]. Consistent with these findings, G0S2 was found to be a target of all-trans retinoic acid (ATRA) in human acute promyelocytic leukemia (APL) cells [38]. ATRA treatment induces terminal differentiation of APL cells and is known to be a highly effective therapy against this form of leukemia (reviewed in [39]). Both mRNA and protein levels of G0S2 were rapidly induced in APL cell line, NB4, and in APL transgenic mice treated with ATRA [38]. In our current study we demonstrate that induction of G0S2 expression in NB4 cells results in elevated TG levels that are likely due to inhibition of ATGL. In addition, knockdown of G0S2 attenuated the effects of ATRA and inhibition of ATGL alone was able to inhibit the growth of NB4 cells. Although several binding partners have been shown for G0S2 including Bcl2 [22], ATGL [19] and Nucleolin [29], our studies suggest that inhibition of ATGL can attenuate cell growth in a wide range of tumour cells and is an important mechanism of G0S2-mediated growth inhibition.

Impressively, over the past few months there has been a succession of four papers independently describing the phenotype of a G0S2 knockout mouse [31-33, 40]. For the most part, each of these studies support the view that G0S2 is a key regulator of lipolysis *in vivo*. G0S2 knockout mice are lean, resistant to obesity by high fat feeding, glucose tolerant, insulin sensitive, and are more thermogenic. Although no studies have thus far reported experiments examining tumour growth in these mice, considering the tumour suppressor properties that the protein appears to have, such experiments would be informative. Studies using knockout mice of ATGL, however, have already revealed interesting insights into cancer biology. ATGL knockout mice were shown to be resistant to cancer cachexia indicating that complex though poorly understood interactions exist between lipid catabolism and tumor biology [41].

## MATERIALS AND METHODS

### Cell lines and drugs

A549, HOP62 and HOP92 and NB4 cells were maintained in RPMI-1640 (Wisent Inc., QC, Canada). HT29 *cells* were maintained in Dulbecco's modified Eagle medium (Wisent Inc., QC, Canada). All media were supplemented with 10% fetal bovine serum (HyClone; Thermo Fisher Scientific Inc.) and 0.1% gentamicin (Wisent Inc., QC, Canada). 1 μM ATRA (Sigma-Aldrich) was added to induce differentiation of NB4 cells. Oleic acid-BSA was purchased from Sigma-Aldrich Co.

### Lentivirus

shRNAs used in this study were expressed from the pLKO vector and were all obtained from Sigma-Aldrich. Identifiers for each of the vectors are as follows: sh-ATGL-1 (NM_020376.2-721s1c1), sh-ATGL-2 (NM_020376.2-718s1c2), sh-G0S2-1 (NM_015714.2-712s1c1), sh-G0S2-2 (NM_015714.2-708s1c1), and SHC002 non-silencing control. Lentiviruses were produced by cotransfection of 293T/17 cells using calcium phosphate coprecipitation with pLKO (shRNA vectors), pCMV-dR8.2 dVPR (Addgene plasmid # 8455), and pCMV-VSV-G (Addgene plasmid # 8455) as described previously [42, 43]. The virus-containing supernatant was then collected, filtered through a 0.22-μm filter, and stored as aliquots at −80°C until further use. Adherent cells were infected with lentivirus on 60-mm dishes. 24 hours after plating, 300 μl virus-containing medium was added in the presence of 10 μg/ml Polybrene. 24 hours post infection the cells were trypsinized and replated in 10-cm dishes. 24 hours after plating, the cells were selected for 48 hours in 1 μg/ml puromycin (Wisent Inc., QC, Canada) before being used for experiments. NB4 cell infections were performed in sterile 15 ml conical tubes by adding 0.5 ml of lentivirus supernatant with 10 μg/ml Polybrene. Cells were centrifuged at 2000 RPM for 1.5 hours at 37°C. Virus-containing medium was aspirated and 2ml of fresh media was added to the cells and plated in a 6 well plate. After 24 hours, cells were selected in media containing 1 μg/ml puromycin.

### Retrovirus

The open reading frame of G0S2 was subcloned from the previously described vector, pCDNA3-G0S2 [22], and ligated into the retroviral vector pBABE including a FLAG epitope tag. Retrovirus particles were produced by cotransfection of 293T cells with pBABE vectors, pCL-Eco and pCMV-VSV-G (Addgene plasmid # 8454). The virus-containing supernatant was collected and stored at −80°C until further use. To establish polyclonal cells stably expressing G0S2, HOP62 and A549 cells were seeded and infected as described above for lentivirus infection.

### Apoptosis assays

Polyclonal cell populations stably expressing shRNAs against ATGL or non-silencing control cells; or stably expressing G0S2-Flag or Empty Vector-Flag, were plated on 6 well plates. The cells were then treated without vehicle; control or with 5FU (100 μM), Camptothecin (10 μM) or Etoposide (25 μM) as indicated in the figure legends. Drugs were purchased from Sigma Aldrich. The cells were stained by washing once with PBS and once with AnnexinV binding buffer (2.5 mM CaCl_2_, 140 mM NaCl, 7.75 mM HEPES [pH 7.4]) and then incubated with 200 μl AnnexinV binding buffer containing 5 μl AnnexinV (BD Biosciences) and 0.25 μg 7-amino-actinomycin D (7AAD) (A.G. Scientific). The cells were analyzed on a Cell Lab Quanta SC flow cytometer (Beckman Coulter).

### Antibodies and western blotting

Anti-FLAG M2 (Cat# F1804), tubulin (Cat# T5168) and actin (Cat# A2066) antibodies were obtained from Sigma-Aldrich. The rabbit anti-ATGL antibody was obtained from Cayman Chemicals. Anti-p53 (DO-1) was purchased from Santa Cruz Biotechnology. Anti-Bcl-XL (Cat# 2762S), Anti-AMPK (Cat# 2532) and phospho-AMPK (Cat# 2535S) were obtained from Cell Signaling. The rabbit polyclonal antibody against G0S2 has been described previously [44]. Whole-cell extracts were obtained by harvesting cells and boiling in 1X Laemmli buffer. Western blotting was performed using standard protocols for SDS-PAGE and wet transfer onto nitrocellulose membranes (Bio-Rad).

### Measurement of intracellular TG content

Triglyceride accumulation was measured using a Triglyceride Quantification Colorometric/Fluorometric kit from BioVision Incorporated. Briefly, 10 million cells were homogenized in a solution containing 5% NP-40 in water. The samples were heated at 100°C until the NP-40 becomes cloudy and cooled down after to room temperature. Another heating cycle was repeated to solubilize all triglyceride. Samples were briefly centrifuged to remove any insoluble material and diluted 10 fold with water before assaying. The samples were measured using the manufactures protocol. The sample O.D. was measured at 570nm in a microtiter plate reader. TG concentration was calculated from a standard curve for each assay. Data shown is normalized to total cellular protein.

### Scratch assays

HT29 cells stably expressing G0S2 shRNAs or non-silencing control (sh-NS) were plated to create a confluent monolayer on a 6 well plate. The monolayer was scratched with a p200 pipet tip and the edges were smoothed by washing the cells once with growth medium. Cells were cultured at 37°C and time lapse photographs taken over two days at several points in each scratch using a Zeiss Axiovert fluorescence microscope. The results are represented as percentage of distance invaded. If width of the scratch at day 0 = D0, width of scratch at day 2 = D2, then percentage of distance invaded = D0-D2/D0 × 100. For the ATGListatin experiment, the scratch was made in a confluent HT29 cells grown on a 6 well plate. 100 μM of ATGListatin or DMSO (control) was added to the cells and cultured for four days.

### Immunoprecipitation

A polyclonal population of A549 cells stably expressing G0S2-Flag or empty vector control (EV) were trypsinized and washed with cold PBS. The cells were lysed in a buffer containing 50mM Tris-HCl (pH 8.0), 135mM NaCl, 10 mM NaF, 1% NP-40, 1.0 mM EDTA, 5% glycerol and protease inhibitor (Roche Diagnostics, Laval, QC, Canada) on ice for 20 min. Cell debris was pelleted by centrifugation and the supernatant was then incubated with 30 μl EZview red anti-FLAG M2 affinity gel (Sigma-Aldrich) for 2 h at 4°C. The beads were then pelleted and washed four times with lysis buffer. The beads were pelleted and resuspended in 1×Laemmli sample buffer and boiled for 5 min prior to immunoblotting analysis.

### Cell growth assays

For all the proliferation assays, equal number of cells were seeded in 12-wells plates and counted at the indicated time points. Trypan blue was used to distinguish live cells from dead ones. The growth curves are shown as an average of 3-4 biological replicates.

### xCELLigence real-time cell analysis (RTCA)

Experiments were carried out using the xCELLigence RTCA DP instrument (Roche Diagnostics GmbH, Mannheim, Germany) which was placed in a humidified incubator at 37°C and 5% CO2. Invasion assays were performed using CIM-16 plates (Roche Diagnostics GmbH, Mannheim, Germany) according to the manufacture protocols. 5×10^4^ cells were seeded in the upper chamber and each experimental condition was performed in quadruplicate with a programmed signal detection every five minutes over 72 hours of incubation. Experiments measuring cytotoxicity of Atglistatin were performed using E-plates (Roche Diagnostics GmbH, Mannheim, Germany) according to the manufacture's protocols. 5000 HOP62 or 4000 A549 cells undergoing exponential growth were seeded in each well. Cell Index value (CI) of each well was monitored every 15 min. For drug treatment, 22 hours after seeding, cells were treated over a period of 72 hours with the indicated concentrations of Atglistatin (Cayman Chemical Company, MI, USA). All data was recorded using the supplied RTCA software (vs. 1.2.1). CI-data from cell growth experiments have been normalized using the RTCA software prior to the start of treatment.

### Statistics

All data is presented as the mean ±SEM. Where indicated statistical testing was performed using the 2-tailed *t*-test on 3 or more replicates. (* indicates *p* ≤ 0.05, **p ≤ 0.01, ***p ≤ 0.001).
